# Novel *GUCA1A* Mutations Suggesting Possible Mechanisms of Pathogenesis in Cone, Cone-Rod, and Macular Dystrophy Patients

**DOI:** 10.1155/2013/517570

**Published:** 2013-08-14

**Authors:** Kunka Kamenarova, Marta Corton, Blanca García-Sandoval, Patricia Fernández-San Jose, Valentin Panchev, Almudena Ávila-Fernández, Maria Isabel López-Molina, Christina Chakarova, Carmen Ayuso, Shomi S. Bhattacharya

**Affiliations:** ^1^Department of Cellular Therapy and Regenerative Medicine, Andalusian Centre for Molecular Biology and Regenerative Medicine (CABIMER), ‘Isla Cartuja', 41092 Seville, Spain; ^2^Department of Genetics, IIS-Jiménez Díaz Foundation, 28040 Madrid, Spain; ^3^Centre for Biomedical Network Research on Rare Diseases (CIBERER), ISCIII, 46010 Valencia, Spain; ^4^Department of Ophthalmology, Hospital ‘Fundación Jiménez Díaz', 28040 Madrid, Spain; ^5^Department of Genetics, UCL-Institute of Ophthalmology, 11-43 Bath Street, London EC1V 9EL, UK

## Abstract

Here, we report two novel *GUCA1A* (the gene for guanylate cyclase activating protein 1) mutations identified in unrelated Spanish families affected by autosomal dominant retinal degeneration (adRD) with cone and rod involvement. All patients from a three-generation adRD pedigree underwent detailed ophthalmic evaluation. Total genome scan using single-nucleotide polymorphisms and then the linkage analysis were undertaken on the pedigree. Haplotype analysis revealed a 55.37 Mb genomic interval cosegregating with the disease phenotype on chromosome 6p21.31-q15. Mutation screening of positional candidate genes found a heterozygous transition c.250C>T in exon 4 of *GUCA1A*, corresponding to a novel mutation p.L84F. A second missense mutation, c.320T>C (p.I107T), was detected by screening of the gene in a Spanish patients cohort. Using bioinformatics approach, we predicted that either haploinsufficiency or dominant-negative effect accompanied by creation of a novel function for the mutant protein is a possible mechanism of the disease due to c.250C>T and c.320T>C. Although additional functional studies are required, our data in relation to the c.250C>T mutation open the possibility that *trans*acting factors binding to de novo created recognition site resulting in formation of aberrant splicing variant is a disease model which may be more widespread than previously recognized as a mechanism causing inherited RD.

## 1. Introduction

Autosomal dominant cone and cone-rod dystrophies (adCODs and adCORDs, resp.) are hereditary retinal disorders characterized by visual loss, abnormalities of color vision, visual field loss, and a variable degree of nystagmus and photophobia. CORDs are characterized by progressive loss of cone photoreceptor function followed by progressive loss of rod photoreceptor function and are often accompanied by retinal degeneration. In contrast, in inherited progressive CODs, only cone function is impaired, and retinal degeneration is often restricted to the central retina [1, 2]. 

COD and CORD are genetically heterogeneous disorders with phenotype caused by mutations in currently ten genes, including *AIPL1*, *CRX*, *GUCA1A*, *GUCY2D*, *PITPNM3*, *PROM1*, *PRPH2/RDS*, *RIMS1*, *SEMA4A*, and *UNC119* (https://sph.uth.edu/retnet/). Four loci (*CORD4*, *RCD1*, *CORD12*, and *CORD17*) with still unidentified genes have been also linked to adCOD and adCORD (https://sph.uth.edu/retnet/). 

*GUCA1A* gene encodes one of three human guanylate cyclase-activating proteins (GCAPs), GCAP1, which is expressed in photoreceptors in the human retina, with stronger GCAP1 immunoreactivity in cones than in rods [12]. A hallmark of the GCAP structure is high affinity Ca^2+^-binding sites, called EF-hands, consisting of helix-loop-helix secondary structure that is able to chelate Ca^2+^ ions [13]. Excitation of the photoreceptor decreases the intracellular concentration of cyclic guanosine-3′,5′-monophosphate (cGMP) and Ca^2+^. GCAP1 works as a Ca^2+^-sensor protein that detects changes in Ca^2+^ concentration and stimulates membrane bound retinal guanylate cyclases (RetGCs) in a Ca^2+^-dependent manner. As a result, the cGMP level is restored [14]. Since its identification, ten disease-causing mutations in the *GUCA1A* gene have been reported to cause autosomal dominant COD, CORD, and macular dystrophy (adMD) in several unrelated families: p.P50L, p.E89K, p.Y99C, p.D100E, p.N104K, p.T114I, p.I143NT, p.L151F, p.E155G, and p.G159V [3–11] (Table 1).

In recent years, the importance of aberrant splicing as a disease mechanism has changed from a situation in which this possibility was considered to be relatively rare and only relevant when one out of a few critical nucleotides in the splice sites consensus sequences at the exon/intron borders are changed to a situation where sequence variants in a gene may potentially disrupt splicing. Thus, in addition to the splice site sequences, other *cis*-regulatory elements which act as splicing enhancers (exonic and intronic, ESEs, and ISEs) or silencers (exonic and intronic, ESSs, and ISSs) and direct the splicing machinery to use the correct splice sites should be considered as important mutation spots [15]. The interactions between silencers, enhancers, and their cognate binding proteins play a critical role in the fidelity and regulation of pre-mRNA splicing [16]. To our knowledge, at least 10% of all mutations identified as causing human-inherited disease are known to alter consensus 5′- or 3′-splice sites, thereby inducing aberrant pre-mRNA splicing [17]. Although the mechanistic consequences of mutations on splice sites are fairly easy to interpret, evaluating precisely how inherited disease-causing mutations influence the loss or gain of ESE/ESS motifs remains challenging [18].

In this study, we report two Spanish families with an autosomal dominant retinal degeneration in which two novel *GUCA1A* mutations have been identified (Table 1). We investigated the relationship between the newly reported coding sequence mutation (c.250C>T) and splicing regulation using a set of bioinformatics techniques and predicted that ESE loss and ESS gain might be responsible for retinal dystrophy. Furthermore, we identified a range of retinal abnormalities within a single pedigree caused by novel GCAP1 p.L84F variation which is consistent with the broader phenotype associated with p.Y99C and p.P50L *GUCA1A* mutations [5, 19].

## 2. Materials and Methods

### 2.1. Clinical Assessment and Sample Collection

**Figure 1:**
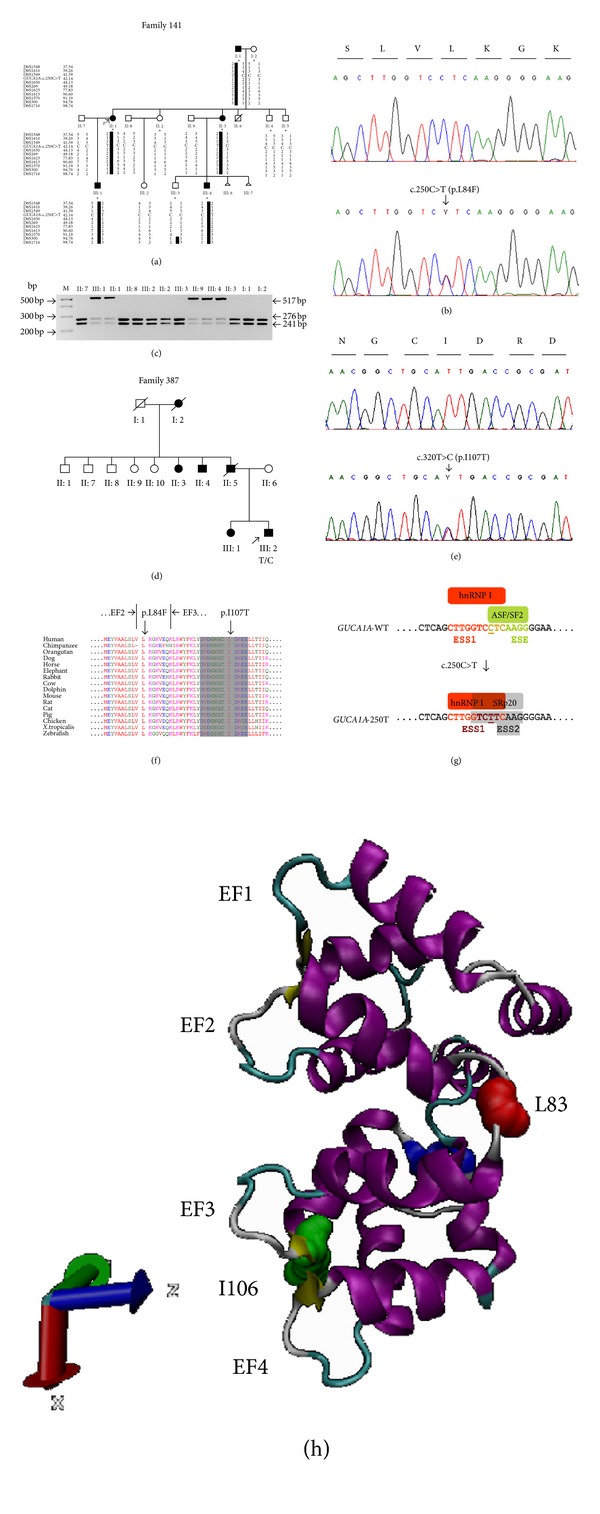
Identification of *GUCA1A* mutations in two Spanish pedigrees. (a), (d) Pedigrees of two unrelated families affected by adRD. Individuals are identified by pedigree number. Squares indicate males, circles indicate females, slashed symbols indicate deceased, solid symbols indicate affected individuals, open symbols indicate unaffected individuals, and arrow indicates the proband. In (a), pedigree of the Family 141 is shown with haplotypes of STR markers spanning the linked interval on chromosome 6. Markers and their physical positions (Mb) are indicated at the left of each row. Ten members indicated with asterisks were genotyped by SNP markers. C/C indicates two copies of wild-type *GUCA1A*, and C/T indicates one copy of wild-type and one copy of mutant *GUCA1A*. In (d), T/C indicates one copy of wild-type and one copy of mutant *GUCA1A* found in the proband III:2 in Family 387. (b), (e) Sequencing chromatograms showing the comparison of DNA sequences of normal control (top) to the heterozygous C-to-T transition in exon 4 of *GUCA1A* (bottom) resulting in a leucine-to-phenylalanine change (p.L84F; GenBank accession number JQ924784) at position 250 (b) and to the heterozygous T-to-C transition in exon 4 of *GUCA1A* (bottom) resulting in a isoleucine-to-threonine change (p.I107T; GenBank accession number JQ924785) at position 320 (e). These mutations segregated with the disease phenotype and were not found in 200 normal controls. (c) Restriction fragment length analysis confirmed the c.250C>T mutation showing that the transition from C to T results in the loss of restriction site of *Sml*I. Wild-type samples produced two fragments of 276 bp and 241 bp, while the restriction target site (5′-**C**TCAAG-3′) in exon 4 of *GUCA1A* was destroyed by the mutation. Analyzed individuals are identified by pedigree number, bp: base pair, and M: 100 bp DNA ladder. (f) Multiple amino acid alignment of known vertebrate showing the evolutionary conservation of guanylate cyclase-activating proteins (only the region containing EF2 and EF3 are shown). Amino acid residues are colored according to the similarity of their physicochemical properties. The conserved 12-amino-acids Ca^2+^-binding loop of EF3 is highlighted in grey. The two mutations occurring in high conserved amino acid region across the species are localized next to EF2 (p.L84F) and within EF3 (p.I107T). (g) The region comprising the exonic splicing enhancer (ESE) and silencer (ESS) motifs in the wild type (WT) exon 4 is compared to the corresponding region in the mutant (250T) exon 4 of *GUCA1A*. The SF2/ASF binding ESE as well as two ESS recognition sites for hnRNP I (ESS1) and SRp20 (ESS2) are depicted. In the context of *GUCA1A* exon 4, we have hypothesized that a binding ESE is disrupted in the case of 250T allele and thus the antagonizing function of SF2/ASF is abolished. Instead, cryptic ESS sites for binding of hnRNP I and SRp20 exon inclusion suppressors are activated which results in exon 4 skipping. The strengthened motif TCTT binding hnRNP I is indicated in dark red. c.C250T substitution is depicted by underlying of the nucleotide. (h) Cartoon representation of chicken Gcap1 (PDB ID: 2R2I) with the Ca^2+^ binding EFhands rendered by VMD software. Residues affected by mutation in the human orthologue are colored in red for Leu84 (corresponding to Leu83 in chicken Gcap1) and green for Ile107 (corresponding to Ile106 in chicken Gcap1).

This study adhered to the tenets of the Declaration of Helsinki for research involving human subjects. A three-generation Spanish family with an autosomal dominant retinal dystrophy was ascertained (Figure 1(a)). Five affected and 9 unaffected individuals were recruited by the Hospital Fundación Jimenez Diaz, Madrid, Spain. After informed consent was obtained, each patient was assessed through a funduscopy, an optical coherence tomography (OCT) using macular cube scan (512 × 128 protocol), an electrooculogram (EOG), a full-field electroretinogram (ERG), and a multifocal ERG (mfERG) using standard electrophysiologic methods. Peripheral blood was obtained by venipuncture, and genomic DNA was extracted from lymphocytes according to standard protocols [20].

A cohort of 61 unrelated Spanish patients clinically diagnosed with COD, CORD, or MD and family history consistent with an autosomal dominant mode of inheritance were additionally screened for mutations in *GUCA1A* gene.

In addition, 200 randomly selected DNA samples (400 chromosomes) from healthy control individuals obtained from the European Collection of Cell Cultures (Human Random Control DNA Panels, ECACC, Salisbury, UK) were analyzed to assess the frequency of sequence changes in the normal population. 

### 2.2. Genotyping and Linkage Analysis

Genotyping of 10 members of the Spanish pedigree (Family 141, Figure 1(a)) was performed with 262,264 single nucleotide polymorphisms (SNPs) from the Affymetrix Gene Chip Mapping 250K Nsp Array (AROS Applied Biotechnologies, Aarhus, Denmark). LOD scores were calculated for the markers by multipoint linkage analysis using Genehunter program in the easyLINKAGE Plus package v5.08 [21]. The call rate, defined as the percentage of successful genotype calls among subjects, was used as a measure of data quality. The results reported in this study are based on 95% call rate. Uninformative SNPs were removed from the data by Merlin [22]. We modeled the disease as an autosomal dominant trait with 100% penetrance. 

All regions with suggestive LOD scores (>1.5) were additionally saturated with microsatellite (STR) markers designed according to the information obtained from Marshfield, GDB Human Genome Database (http://research.marshfieldclinic.org/) and Ensembl genome data resources (http://www.ensembl.org/). Multiplex microsatellite genotyping of all available family members (Figure 1(a)) was performed with 3–5 markers per reaction using QPCR mix (ABgene, UK). Data collection and allele identification were performed using GeneScan and GeneMapper v4.0 software (Applied Biosystems Incorporated, Foster City, CA, USA).

### 2.3. Mutation Screening

Sequence analysis of the coding exons and intron-exon junctions of candidate genes within the critical interval, *IMPG1, ELOVL4*, *RDS*, *GUCA1A*, and *RIMS1,* was performed on ABI3730xl genetic analyser (Applied Biosystems Incorporated, Foster City, CA, USA) according to the manufacturer's protocol. Novel nucleotide sequences were compared to the published cDNA sequence according to the human genome build 37 (hg19, 2009). 

### 2.4. Restriction Enzyme Analysis

To confirm the presence of c.250C>T variant, exon 4 of the human *GUCA1A* gene was directly amplified from genomic DNA. A sample of genomic DNA (100 ng) from patients and healthy individuals was used in a 20 *μ*L reaction mixture containing 0.5 *μ*M of forward (5′ GTCTAGGAAGACAGATAGGTC 3′) and reverse (5′ CAAGGAAGGGAAGGAACGTG 3′) primers, 20 *μ*M of each dNTP, 1× PCR buffer, and 1 U of MyTaq DNA polymerase (Bioline, London, UK). After an initial denaturation of 95°C for 5 min, 30 cycles were performed at 95°C for 30 s, 62°C for 20 s, and 72°C for 50 s, with a final extension step of 72°C for 5 min. The PCR products (517 base pairs length) were digested with *Sml*I according to the manufacturer's instructions (New England BioLabs, Beverly, MA, USA) and separated by electrophoresis in a 3% agarose gel. 

### 2.5. Mutation Prediction and In Silico Analyses of ESE and ESS Motifs

Prediction of the possible effect of an amino acid substitution on the structure and function of the human protein was performed with the following software tools. PolyPhen-2 (http://genetics.bwh.harvard.edu/pph2/) generates a different scale of reported scores, where a score of 0–0.2 is considered “benign,” 0.2–0.85 is “possibly damaging,” and 0.85–1.0 is “probably damaging” [23]. PSIPRED (http://bioinf.cs.ucl.ac.uk/psipred/) is a secondary structure prediction method with a 80% accuracy according to the (CASP Critical Assessment of Structure Prediction techniques) contents [24], incorporating two feed-forward neural networks which perform an analysis on output obtained from PSI-BLAST (Position-Specific Iterated-BLAST) [25]. Computer programs, Human Splicing Finder 2.4.1 (http://umd.be/HSF/) [26] and ESEfinder 3.0 (http://rulai.cshl.edu/cgi-bin/tools/ESE3/esefinder.cgi) [27], were used for prediction of ESE and ESS motifs present in the wild-type or mutant *GUCA1A* exon 4 sequence. Alternative Splicing Database (ASD), Splicing Rainbow (http://www.ebi.ac.uk/) [28], and the ESRsearch web server (http://esrsearch.tau.ac.il/) [29] were utilized for searching of both ESE and ESS putative elements indicated by the RESCUE-ESE [30], FAS-ESS [31], and (PESX Putative Exonic Splicing Enhancers/Silencers) [32] servers. 

### 2.6. Protein Structure Modeling

Chicken (*Gallus gallus*) orthologue Gcap1 (PDB ID: 2R2I) was modeled using Visual Molecular Dynamics (VMD) software (http://www.ks.uiuc.edu/Research/vmd/) [33].

## 3. Results

### 3.1. Clinical Evaluation of Patients

**Figure 2:**
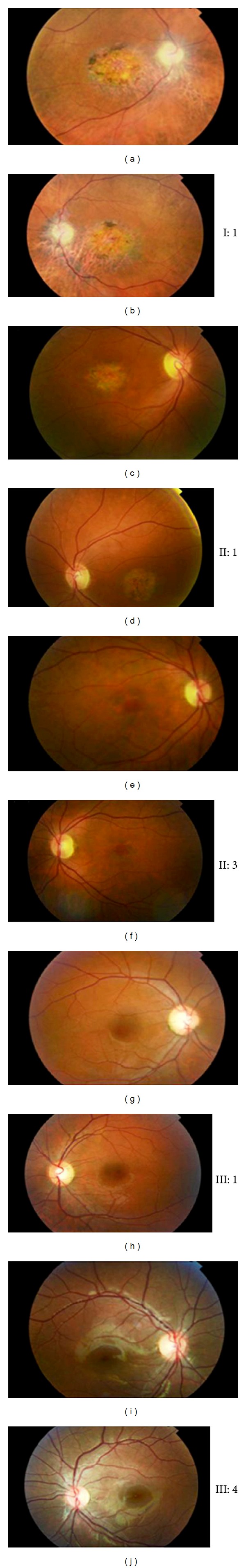
Color fundus photographs of both eyes of patients carrying the mutation p.L84F in *GUCA1A*. (a), (b) Patient I:1 (75 y). Fundus photographs showing an evident disc pallor and arteriolar attenuation, area of macular atrophy with pigment migration, choriocapillaris atrophy surrounded this area, and the optic disc bilaterally. (c), (d) Patient II:1 (53 y). Fundus photographs showing bilateral temporal disc pallor, mild arteriolar attenuation, and bull's eye pattern of maculopathy. (e), (f) Patient II:3 (47 y). Funduscopy showing mild temporal disc pallor, normal vessels, and perifoveal retinal pigment epithelium (RPE) alterations bilaterally. (g), (h) and (i), (j) Patients III:1 (25 y) and III:4 (12 y), respectively, presenting normal fundi.

**Figure 3:**
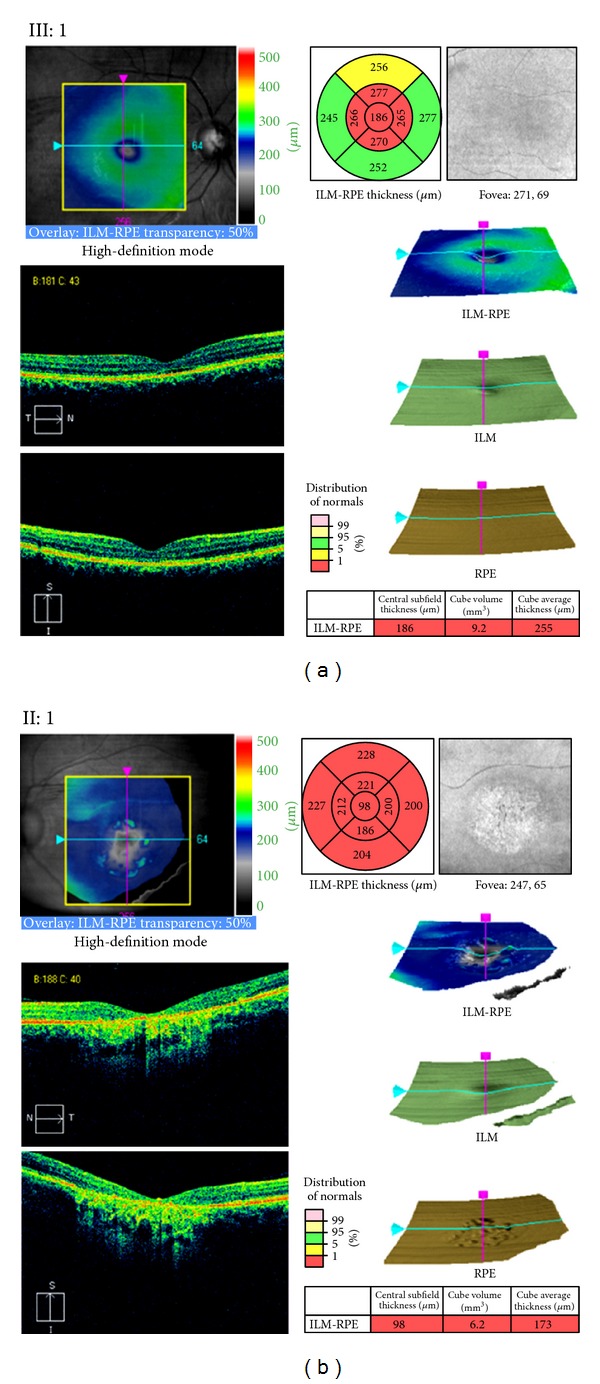
Optical coherence tomography (OCT) macular cube 512 × 128 scan showing macular thickness in individuals III:1 (a) and II:1 (b). For each scan, top left: fundus image with scan cube overlay. Top right: macular thickness significance map. The central innermost 1-mm-diameter circle represents the central subfield; inner superior, inner nasal, inner inferior, and inner temporal areas bounded by the 3-mm-diameter circle form the inner macula; outer superior, outer nasal, outer inferior, and outer temporal areas bounded by the 6-mm-diameter circle form the outer macula. Retinal thickness values from ILM to RPE are compared to the normative data. Middle and bottom left: cross-sectional OCT scans. Middle right: 3D surface maps: the ILM-RPE, displaying the retinal thickness in three dimensions, the ILM, which appears normal in the two patients, and the RPE, which is altered in II:1. Bottom right: central subfield thickness, overall average macular thickness, and overall macular volume compared to normative data are displayed in table format. Reduction in the retinal thickness and macular atrophy of all retinal layers occurs in patient II:1. Abnormally thin macular region appears even in patient III:1 before obvious ophthalmoscopic changes. ILM: inner limiting membrane; RPE: retinal pigment epithelium.

**Figure 4:**
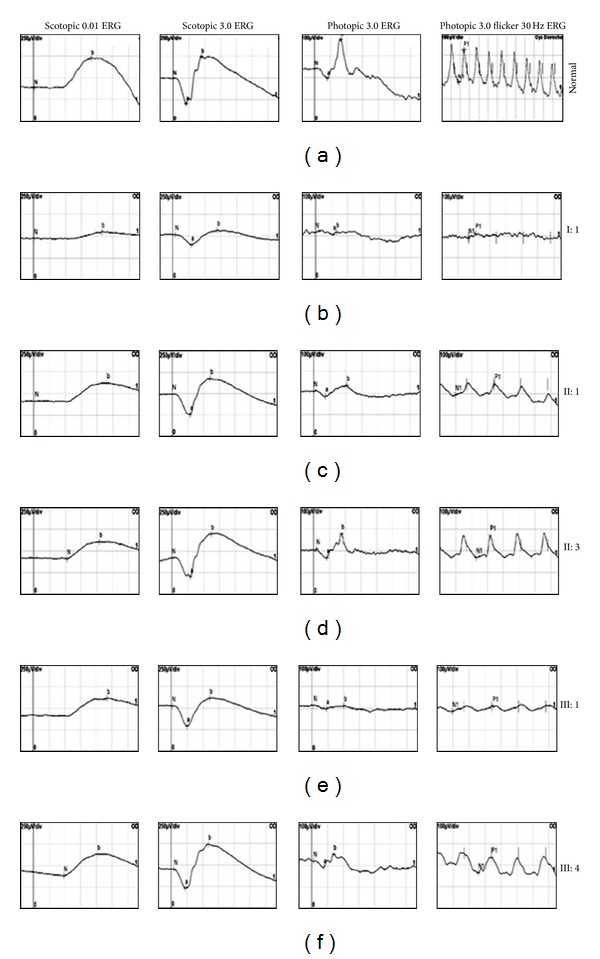
Right eye flash electroretinograms (ERGs) in patients I:1 (b), II:1 (c), II:3 (d), III:1 (e), and III:4 (f), carrying the mutation p.L84F, in comparison with ERG of a representative control subject (a). Dark-adapted ERGs are shown for flash intensities of 0.01 and 3.0 cd·s/m^2^; light-adapted ERGs and 30 Hz flicker ERGs are shown for a flash intensity of 3.0 cd·s/m^2^. The electrophysiological findings are consistent with cone-rod dystrophy with absent photopic response and decreased scotopic response in subject I:1 (b) or cone dystrophy showing photopic ERG with decreased photopic amplitudes in patients II:1, III:1, and III:4 ((c), (e), and (f)). Despite the progressive loss of visual acuity observed in individual II:3, photopic ERG shows normal amplitudes (d). Implicit time of the scotopic response was normal whereas delayed photopic responses were observed in all patients.

**Figure 5:**
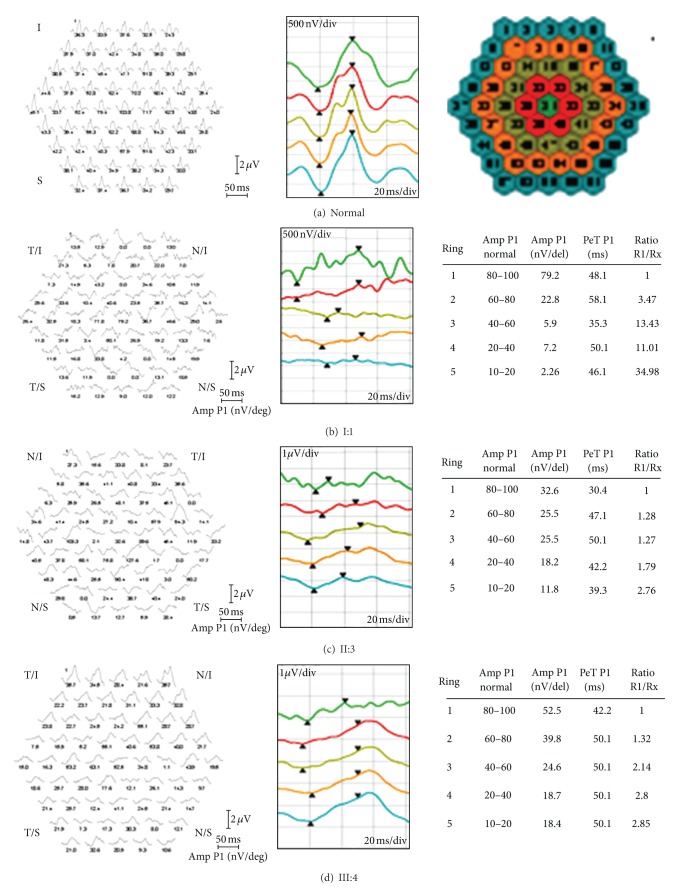
Right eye multifocal ERG (mfERG) in individuals I:1 (b), II:3 (c), and III:4 (d) compared with mfERG of a representative control subject (a). mfERG records reduced to nonrecordable central function. The ring analysis (see schematic) goes from the center to the periphery. Quantitative results of mfERG analyses are displayed in table format.

Clinical evaluation including fundus photography and electrophysiology was performed in a three-generation Spanish pedigree with autosomal dominant retinal degeneration with cone and rod involvement (Family 141, Figure 1(a)). All affected individuals complained of very early loss of central vision within the first two decades (between age of 2 and 17 years), with subsequent gradual deterioration of the color vision and mild photophobia. There was no evidence of nystagmus. Clinical characteristics of patients are summarized in Tables 2 and 3. Funduscopy revealed a range of macular phenotypes, from normal or mild changes to severe macular atrophy (Figures 2(a)–2(j)). This variation was not related to the age of the subjects. Despite the normal fundus appearance in patient III:1, OCT scans showed that the macular region was abnormally thin. However, retinal layers at the macular area were still well conserved in this subject (Figure 3(a)). Instead, the affected mother (subject II:1) clearly presented disorganization of the retinal layers at the macular region, and reduction in the retinal thickness also appeared (Figure 3(b)). In the presence of common genotype, electrophysiologic examination of the patients detected intrafamilial variability of the phenotype involved either the cones only or both cone and rod systems with photopic amplitudes more markedly reduced than the scotopic (Figure 4). The oldest subject (individual I:1) had bilaterally undetectable cone response and subnormal rod ERGs (Figure 4(b)), indicating a cone-rod phenotype with severe loss of macular function (Figure 5(b)). Patients II:1, III:1, and III:4 all presented from moderate (III:4) to significantly reduced (II:1 and III:1) cone ERGs and normal rod responses on full-field ERG (Figures 4(c), 4(e), and 4(f)), suggestive of cone dysfunction. Additional evidence for macular dysfunction is presented as detected by mfERG (Figure 5(d)) and OCT imaging (Figure 3). In patient II:3, the full-field cone and rod ERGs were normal in both eyes (Figure 4(d)), but the mfERG showed decreased central amplitudes reflecting the macular dysfunction (Figure 5(c)); findings are consistent with isolated severe bilateral macular degeneration and resulted in a loss of the central vision. No abnormalities were observed in the EOGs in any patient (Table 2). All affected individuals studied in detail were later found to carry the p.L84F *GUCA1A* mutation.

### 3.2. Genotyping and Linkage Analysis

Five affected and nine unaffected subjects of the Spanish family (Figure 1(a)) were genotyped with Affymetrix 250K SNP microarray, and genome-wide linkage analysis was used to search for cosegregation of the markers with the disease phenotype. Parametric multipoint analysis using Genehunter revealed a 55.37 Mb genomic interval between rs2894403 (35,507,179 bp) and rs2474618 (90,876,844 bp) with a maximum likelihood odds ratio score of 2.98 at a recombination fraction 0.0 suggestive for linkage. Secondary peaks generating LOD score of 2.11 were observed for five different intervals on chromosomes 1, 7, 11, 12, and 16. Consequently, additional densely spaced microsatellite markers spanning these regions were used and haplotypes were constructed. Nonsegregation of STR-haplotypes within the family allowed exclusion of all intervals except the interval located on chromosome 6 which could not be further refined. Affected individuals shared a common haplotype tested with microsatellite markers spanning the linked region and generating maximum LOD score of 2.10 at *θ* = 0.0 (Figure 1(a)). 

### 3.3. Mutation Analysis

According to Ensembl human genome database, the 55.37 Mb critical interval on chromosome 6p21.31-q15 included 295 known genes among which were previously described retinal degeneration genes. Positional candidate genes were *IMPG1* and *ELOVL4*, mutations in which cause adMD [34, 35]; as well as *RDS*, *GUCA1A*, and *RIMS1*, implicated in autosomal dominant COD and CORD [9, 36, 37]. By direct sequencing of the coding exons and intron-exon junctions of the candidate genes, we found only known polymorphisms except a novel heterozygous substitution c.250C>T in *GUCA1A*, replacing leucine with phenylalanine at amino acid residue 84 (Figure 1(b)). Segregation analysis showed that the novel mutation (p.L84F) co-segregated with the disease in the pedigree (Figure 1(a)), but it was not detected in 200 unrelated normal controls. This variant was not reported in public sequence repositories (including the 1000 Genomes Project database, http://www.1000genomes.org/).

A restriction fragment length analysis confirmed the c.250C>T mutation showing that the transition from C (wt) to T (mutation) results in loss of *Sml*I restriction site. Wild-type samples produced two fragments of 276 bp and 241 bp, while the restriction target site (5′-**C**TCAAG-3′) in exon 4 of *GUCA1A* was destroyed by the mutation (Figure 1(c)). 

A second missense mutation c.320T>C (p.I107T), in exon 4 of *GUCA1A*, was found in an index case (Family 387, individual III:2) after screening a cohort of 61 patients (Figures 1(d) and 1(e)). No DNA was available from other family members for testing of the cosegregation, but a family history of adRD was reported (Family 387, Figure 1(d)). Moreover, the variant was not found in 400 chromosomes from ethnically matched control individuals and was novel according to 1000 Genomes Project data. The clinical findings in this family were very similar to the complex phenotype of Family 141 associated with the p.L84F GCAP1 mutation, and major hallmarks are summarized in Table 2.

### 3.4. Mutation Prediction

Both missense mutations, p.L84F and p.I107T (GenBank accession numbers JQ924784 and JQ924785, resp.), were located in amino acid residues well conserved across different species (Figures 1(f) and 1(h)). By PolyPhen-2 and PSIPRED programs, we further compared the predicted consequences on protein secondary structure of all reported GCAP1 mutations, p.P50L [3], p.E89K [4], p.Y99C [6], p.D100E [4], p.N104K [7], p.T114I, p.I143NT [8], p.L151F [9, 10], p.E155G [11], and p.G159V [4], as well as of the two novel changes, p.L84F and p.I107T. PolyPhen-2 predicted a “probably damaging” effect for the two mutations, with scores of 0.999 and 1.0, respectively (Table 1). PSIPRED correctly predicted protein secondary structure change in all 10 mutations known to cause reduction of retinal guanylate cyclase-1 (RetGC1) activity [3–11] and also for the new variant p.I107T (Table 1). Multiple changes of the protein topology including shortening and/or increasing of *α*-helices and coil stretches (caused by the mutations p.P50L, p.E89K, p.Y99C, p.D100E, p.N104K, p.I107T, p.T114I, p.I143NT, p.L151F, p.E155G, and p.G159V), as well as formation of novel *β*-strands of 2-3 amino acids (caused by the mutations p.P50L, p.E89K, p.Y99C, p.D100E, p.I143NT, p.L151F, p.E155G, and p.G159V) were predicted. Interestingly, PSIPRED predicted structural change for p.I107T at four locations. 12 amino acid *α*-helix (residues 88–99, equivalent to EF3) was shortened by two amino acids, 11 amino acid *α*-helix (residues 132–142, equivalent to EF4) was increased by one amino acid, 7 amino acid *α*-helix (residues 176–182) was increased by one amino acid, and C-terminal 9 amino acid *α*-helix (residues 191–199) was broken into two by one-residue coil at position 193 (data not shown). No structural alterations of the encoded protein were predicted for c.250C>T (p.L84F) suggesting different mechanism of pathogenesis. 

We hypothesized that the c.250C>T mutation could function by disrupting a splicing enhancer and/or by the creation of a splicing silencer because recognition of splice sites can either be assisted by splicing enhancers or suppressed by splicing silencers. Therefore, we scanned the wild-type and c.250C>T mutant *GUCA1A* exon 4 sequence for ESEs and ESSs using different web-based programs. The results generated by ESEfinder 3.0 program indicated that the 250T nucleotide abolishes SF2/ASF binding motif (**C**TCAAGG) (Figure 1(g)). An overlapping SC35 binding motif (GGTC**C**TCA) is also disrupted, while a second SC35 binding site (GTC**C**TCAA>GTC**T**TCAA) is not affected by the mutation, but its score has been decreased from 3.572 to 3.341 (Table 4). Although Human Splicing Finder calculates the score according to a different algorithm [26], the two analyses gave similar results. 

The ASD and ESRsearch programs identified that both ESE (which promotes exon inclusion) and ESS (which removes identified exon from the final product) elements present on *GUCA1A* exon 4. These programs confirmed the loss of SF2/ASF ESE site (**C**TCAAGG) caused by the mutant allele. In addition, the c.250C>T mutation creates a new ESS site for binding of SRp20 (GTC**T**TCAAG) and strengthens the binding motif of the *trans*acting splicing repressor hnRNP I (CTTGGTC**C**TC>CTTGGTC**T**TC). ESRsearch program predicted the mutant TC**T**TC motif as a gain of novel hnRNP I binding ESS (Figure 1(g)). 

## 4. Discussion

In this study, we identified a three-generation Spanish pedigree representing intrafamilial heterogeneity of retinal degeneration with cone and rod involvement. The clinical observations revealed a range of retinal abnormalities, including isolated macular dysfunction, cone dystrophy and cone-rod dystrophy—findings very similar to previous descriptions of the mixed phenotype associated with p.P50L and p.Y99C GCAP1 mutation [5, 19]. The very early onset of visual loss and the fast progression of the retinal dystrophy in the presented family contribute to the wide variability of phenotypes associated with GCAP1 mutations. Combined with previous studies, our findings support the hypothesis of additional modifying factors (genetic and environmental) involvement, which are responsible for the modulation of the phenotype in patients harboring the same *GUCA1A* mutation [5].

Linkage to the *GUCA1A* locus was identified, and two novel missense mutations, p.L84F and p.I107T, predicting different mechanisms of pathogenesis were found in exon 4 of the GCAP1 coding gene (Figures 1(a)–1(e)). The Ile107 residue is located in a phylogenetically conserved motif within the functional EF3 hand domain of the guanylate cyclase-activating protein 1 (Figures 1(f) and 1(h)). The result of PSIPRED analysis suggested that the novel p.I107T mutation led to secondary structure changes affecting EF3, EF4, and two C-terminal *α*-helices which may interfere with the correct folding of the two helix-loop-helix Ca^2+^-binding hands. A number of missense mutations associated with either loss or gain of function and producing COD, CORD, and MD dominant phenotypes have been already discovered. By using PSIPRED, we predicted that each of these mutations (p.P50L, p.E89K, p.Y99C, p.D100E, p.N104K, p.T114I, p.I143NT, p.L151F, p.E155G, and p.G159V) specifically alter the conformation of the protein which is essential for the Ca^2+^ binding (Table 1). 

The p.I107T mutation represents the second naturally occurring mutation in the 12-amino-acid-loop of the third GCAP1 EF hand. Previously, a mutation (p.N104K) affecting the Ca^2+^-binding loop of EF3 has been found to be causative for adCOD [7]. It has been also reported that a mutation of a residue flanking EF3 (p.Y99C) disrupts the N-terminal helix of the helix-loop-helix conformation of EF3, severely affecting Ca^2+^ binding at this site [6]. Another pathogenic mutation of a flanking hydrophobic residue (p.I143NT) was observed in EF4, emphasizing the importance of an intact N-terminal helix for Ca^2+^ binding [8]. Other mutations linked to cone dystrophy (p.E155G and p.L151F) affect exclusively Ca^2+^ coordination in EF4 [9–11]. Common biochemical effect of the mutations affecting one of the functional EF hands consists of the inability of the mutant GCAPs to inhibit photoreceptor GC in the dark when Ca^2+^ is elevated [38]. The result is that cGMP levels are elevated in mutant photoreceptors and a larger number of cation channels remain open, which eventually leads to elevated Ca^2+^ concentration and to photoreceptor cell death [39]. Taken together, all this data and the fact that the mutation is of the missense type suggest similar functional consequence of p.I107T, and the mechanism of disease causation should be considered as either loss or gain of function that affects calcium homeostasis within the photoreceptor outer segment.

The mutation p.L84F is located in another phylogenetically conserved motif flanking the functional EF2 hand (Figures 1(f) and 1(h)). Although PolyPhen-2 predicted probably damaging effect for this mutation, no conformational changes of the mutant protein were shown by PSIPRED program (Table 1). Therefore, we used a set of computational approaches to determine if this substitution alters specific *cis*-acting elements that are important for correct splicing, such as ESEs and ESSs, and acts at an earlier RNA level. 

Two of the major factors in establishing exon identity are serine- and arginine-rich proteins (SR proteins), among which are SF2/ASF and SC35, and the heterogeneous nuclear ribonucleoproteins (hnRNPs) [15]. SR proteins promote the initial stages of spliceosome assembly by binding to ESEs and recruiting basal splicing factors to adjacent splice sites or by antagonizing the effects of ESS elements [40]. In contrast, hnRNPs member among which is hnRNP I (also known as polypyrimidine tract-binding protein, PTB), mediate the repressive effects of silencers and can alter recruitment of the core splicing machinery [41]. Surprisingly, since SR proteins are typically thought to promote exon inclusion, it has been demonstrated that interactions between SRp20, PTB, and other hnRNPs create an exon silencing complex that promotes exon skipping and cause inherited disease [42].

Our in silico analyses of exon 4-wild type and mutant sequence indicated that naturally occurring SF2/ASF (splicing factor 2/alternative splicing factor) ESE motif (CTCAAGG) was abolished (SF2/ASF: 250C score = 2.216, threshold = 1.956) suggesting that this heptamer might be essential to distinguish a functional exon and its disruption results in loss of activity. In addition, it was shown that the c.250C>T mutation disrupted (SC35: 250C score = 3.059, threshold = 2.383) a binding ESE motif (GGTCCTCA) and weakened (SC35: 250C score = 3.572, 250T score = 3.341, and threshold = 2.383) a second recognition site for the RNA-binding protein SC35 (GTC**C**TCAA>GTC**T**TCAA) (Table 4). A search for potential splicing silencer regulatory elements within the *GUCA1A* exon 4 showed that the 250T allele created a new ESS site (GTC**T**TCAAG) for binding of SRp20 which is not present with 250C and strengthened another binding motif (TC**C**T>TC**T**T) for hnRNP I/PTB (Figure 1(g)), both designated as exon silencer partners by Sterne-Weiler and coauthors [42]. It has been shown that the loss of an ESE consensus motif for SF2/ASF can cause human-inherited disease [43]. There are many other examples for disease-associated mutations that alter splicing enhancers or silencers and promote exon skipping, which support our findings [42, 44–48]. Similarly, disease-causing mutations in introns that cause missplicing by inducing the inclusion of intronic sequences as exons (pseudoexon inclusion) and function by strengthening of preexisting weak pseudosplice sites or by creating new splice sites have been discovered [49–51]. 

Based on this data, we concluded that the c.250C>T mutation may function by disruption of SF2/ASF and SC35 binding ESEs and activation of cryptic ESS sites (for binding of hnRNP I and SRp20) which promotes in-frame skipping of exon 4 from the spliced mRNA. Thus, the expected consequence of this mutation is production of aberrant transcript that, if translated, would encode a truncated (50 amino acids shorter) protein copy suggesting either gain of function or haploinsufficiency with cone cells predominantly affected because of the higher concentrations of RetGC1 and GCAP1 in cone photoreceptors.

## 5. Conclusions

In conclusion, we suggest that mutation associated with disruption of ESE and activation of ESS *cis*-elements can be applied to photoreceptor degeneration. Importantly, this means that similar molecular mechanism may cause retinal degeneration much more frequently than reported for aberrant spliced mRNA so far. 

However, because of their predictive nature, these findings need to be validated with experimental evidences in order to see the real impact of the reported missense mutations on the structure and conformation of GCAP1.

## Figures and Tables

**Table 1:** Mutation prediction for known and novel *GUCA1A* variations.

Mutation	Domain	PolyPhen-2^a^		PSIPRED^b^	Dystrophy	Reference
		Prediction	Human var score	Protein secondary structure change		
P50L		Benign	0.132	Yes	Cone, rod-cone	[3]
L84F		PRD	0.999	No	Cone, cone-rod, macular	Novel
E89K	EF3	PSD	0.681	Yes	Cone	[4]
Y99C	EF3	PRD	1.000	Yes	Cone, cone-rod, macular	[5, 6]
D100E	EF3	PRD	0.999	Yes	Cone	[4]
N104K	EF3	PRD	0.998	Yes	Cone	[7]
I107T	EF3	PRD	1.000	Yes	Cone, cone-rod, macular	Novel
T114I	EF3	Benign	0.009	Yes	Atypical RP	[8]
I143NT	EF4	NP	—	Yes	Cone	[8]
L151F	EF4	PRD	1.000	Yes	Cone-rod	[9, 10]
E155G	EF4	PRD	1.000	Yes	Cone	[11]
G159V	EF4	PRD	1.000	Yes	Cone	[4]

^a^PolyPhen-2 appraises mutations qualitatively as benign, possibly damaging (POS), or probably damaging (PRD) based on the model's false positive rate. NP: not performed.

^b^PSIPRED was used for secondary structure prediction, and the number of the resulting alterations is given in the table. For PSIPRED analysis, any predicted changes in protein secondary structure were considered to be damaging mutations.

**Table 2:** Phenotypic characteristics and electrophysiological data of patients with *GUCA1A* mutations.

FamilyID/ PatientID/ *GUCA1A* mutation	First symptoms (age) and course	Age (y)	Most recent phenotype (RE // LE)								
			VA	Refractive error	Visual field	Amplitudes ERG Scotopic // Photopic	Implicit time ERG Scotopic // Photopic	mfERG	EOG	OCT	Anterior segment
141/I:1/ c.250C>T; p.L84F	Loss of VA at early childhood (1-2 y), color vision deficiency, and photophobia. No NB	75	0.05 // 0.05	+0.50 −2.50 90°// +0.50 −2.00 80°	Central scotomas // Central and peripheral scotomas	Low amplitudes // NR Figure 4(b)	Normal // NR Figure 4(b)	NR or diminished responses Figure 5(b)	Normal	NP	Pseudophakia in BE
141/II:1/ c.250C>T; p.L84F	Early progressive loss of VA (17 y), mild color vision deficiency, and photophobia. No NB	53	0.05 // 0.05	+050 −100 165°// +050 −075 15°	Central and peripheral scotomas // Peripheral scotomas	Normal // Low amplitudes Figure 4(c)	Normal // Augmented Figure 4(c)	NR or diminished responses	Normal	Neurosensorial atrophy Figure 3(b)	Normal in BE
141/II:3/ c.250C>T; p.L84F	Early progressive loss of VA (13 y), color vision deficiency, and photophobia. No NB	47	0.1 // 0.1	+0.75 −0.50 130° // +0.75 −0.50 50°	Central and superior scotoma BE	Normal // Normal Figure 4(d)	Normal // Mixed: Augmented Figure 4(d)	Diminished responses Figure 5(c)	Normal	NP	Normal in BE
141/III:1/ c.250C>T; p.L84F	Early progressive loss of VA (6 y), mild color deficiency, and photophobia. No NB	25	0.3 // 0.3	−6.00 −3.50 180° // −5.00 −3.75 180°	Central and peripheral scotomas BE	Normal // Very low amplitudes Figure 4(e)	Normal // Augmented Figure 4(e)	Diminished responses	Normal	Neurosensorial atrophy Figure 3(a)	Normal in BE
141/III:4/ c.250C>T; p.L84F	Early progressive loss of VA (6 y), photophobia. No NB	12	0.4 // 0.4	+0.50 −0.50 110° // +0.50 −0.75 85°	Normal // Central low density scotoma	Normal // Low amplitudes Figure 4(f)	Normal // Augmented Figure 4(f)	Diminished responses Figure 5(d)	Normal	NP	Normal in BE

387/III:2/ c.250C>T; p.L84F	Loss of VA (36 y), color vision deficiency, and photophobia. No NB	38	0.7 // 0.6	NP	Central scotoma	Low amplitudes // Low amplitudes	NP	NP	NP	Sparse RPE alteration at the macula	Normal in BE

RE: right eye; LE: left eye; VA: visual acuity; ERG: electroretinogram; mfERG: multifocal ERG; EOG: electrooculogram; OCT: optical coherence tomography; NB: night blindness; M: male; F: female; NR: nonrecordable; NP: not performed; BE: both eyes; RPE: retinal pigment epithelium.

**Table 3:** Quantitative measurements of ERG findings.

	Scotopic 0.01 ERG		Scotopic 3.0 ERG					Photopic 3.0 ERG				Photopic 3.0 flicker 30 Hz ERG		
	b (ms)	b-wave (*µ*V)	a (ms)	b (ms)	a-wave (*µ*V)	b-wave (*µ*V)	b/a (V)	a (ms)	b (ms)	a-wave (*µ*V)	b-wave (*µ*V)	P1 (ms)	N1-P1 (*µ*V)	30 Hz Amp
Normal range	67–91	95–305	14–22	33–46	155–356	290–654	1.5–2.6	13–16	29–33	26–62	103–250	58–64	57–223	
I:1														
OD	86	74	23	56	116	167	1.4	22	26	10.0	11.3	42	22.7	4.43
OS	85	74	23	53	126	167	1.3	21	26	2.54	5.88	46	13.4	2.58
II:1														
OD	93	211	23	49	238	423	1.8	15	41	23.6	50.9	69	51.6	25.5
OS	87	270	23	48	266	466	1.8	16	41	27.3	60.3	69	65.5	27.9
II:3														
OD	84	199	23	50	218	498	2.3	17	36	41.1	112.0	64	102.0	35.3
OS	78	164	24	50	245	490	2.0	17	35	40.7	121.0	64	110.0	36.1
III:1														
OD	94	81.1	20	49	234	325	1.4	18	41	14.8	14.4	70	25.3	10.1
OS	95	201	21	50	231	341	1.5	18	34	14.4	15.5	69	25.5	9.37
III:4														
OD	82	251	18	47	220	510	2.3	15	28	30.1	62.4	68	72.9	35.5
OS	79	215	18	47	170	434	2.6	15	28	32.2	67.6	67	74.7	27.6

**Table 4:** ESEfinder 3.0 analyses of the wild-type and c.250C>T mutant exon 4 of *GUCA1A*.

Splicing factor mutation	SF2/ASF threshold = 1.956			SC35 threshold = 2.383		
	Position (nt)^a^	Motif	Score	Position (nt)^a^	Motif	Score
Wild type	49	**C**TCAAGG	2.216	45	GGTC**C**TCA	3.059
				46	GTC**C**TCAA	3.572

GUCA1A-L84F	49	—	—	45	—	—
				46	GTC**T**TCAA	3.341

^a^Position of the first nucleotide of an ESE motif counted from the 5′ boundary of exon 4 of *GUCA1A*.
